# Effectiveness of Stretching in Post-Stroke Spasticity and Range of Motion: Systematic Review and Meta-Analysis

**DOI:** 10.3390/jpm11111074

**Published:** 2021-10-24

**Authors:** Laura Gomez-Cuaresma, David Lucena-Anton, Gloria Gonzalez-Medina, Francisco Javier Martin-Vega, Alejandro Galan-Mercant, Carlos Luque-Moreno

**Affiliations:** 1 Department of Nursing and Physiotherapy, University of Cádiz, 11009 Cádiz, Spain; laura.gomezcuaresma@alum.uca.es (L.G.-C.); gloriagonzalez.medina@uca.es (G.G.-M.); javier.martin@uca.es (F.J.M.-V.); alejandro.galan@uca.es (A.G.-M.); carloslm@us.es (C.L.-M.); 2 Intell-SOK (TIC-256) Research Group, Department of Informatics Engineering, University of Cádiz, 11519 Cádiz, Spain; 3 Investigation Group CTS-986, Physical Therapy and Health (FISA), University Institute of Research in Social Sustainable Development (INDESS), University of Cádiz, 11009 Cádiz, Spain; 4 MOVE-IT Research Group, Department of Physical Education, Faculty of Education, Sciences University of Cádiz, 11002 Cádiz, Spain; 5 Biomedical Research and Innovation Institute of Cádiz (INiBICA) Research Unit, Puerta del Mar University Hospital, University of Cádiz, 11002 Cádiz, Spain; 6 Department of Physiotherapy, Faculty of Nursing, Physiotherapy and Podiatry, University of Seville, 41009 Seville, Spain

**Keywords:** stroke, muscle spasticity, muscle stretching exercises, activities of daily living

## Abstract

Spasticity is one of the most frequent and disabling clinical manifestations of patients with stroke. In clinical practice, stretching is the most widely used physiotherapeutic intervention for this population. However, there is no solid evidence for its effectiveness. The aim of this study was to evaluate the effectiveness of different types of stretching in reducing post-stroke spasticity. Research was carried out until March 2021 in the following scientific databases: PubMed, CINAHL, Scopus, Cochrane Library, Web of Science, and PEDro. The PEDro scale and the Cochrane collaboration tool were used to assess the methodological quality and risk of bias of the studies. Eight articles were selected for qualitative analysis; six of them contributed information to the meta-analysis. No conclusive evidence was obtained on the effectiveness of stretching in terms of treating spasticity and range of motion in patients with stroke. Further research is necessary in order to determine the effectiveness of the use of stretching in this population, considering the different types of stretching (static and dynamic), the time of application, the measurement of the different components of spasticity, and the extrapolation of functional results.

## 1. Introduction

A stroke is defined as a sudden focal neurological deficit caused by an abnormality, which depends on the affected area of the brain [1]. It has an incidence ranging from 144 to 187 per 100,000 inhabitants per year, and is one of the top four global causes of death [2]. Aphasia, negligence, sensory loss, pain, dysarthria, cognitive deficits, dysphagia, depression, and, above all, motor weakness are common disorders produced by stroke, all of which can lead to significant limitations [1]. Strokes have long-lasting and profound effects on the patient, with the greatest impact attributable to impaired neurological function [3]. Strokes are one of the main causes of permanent disability, since more than two thirds of survivors develop sequelae, such as spasticity [1,2,3,4,5,6]. When this occurs in the lower extremities, this results in reduced independence [7].

Spasticity is a very common complication in patients with stroke (pwS). It is associated with lesions of the central nervous system, which cause different clinical syndromes, such as spasms or clonus [8]. Spasticity is defined as the hyperexcitability of muscles resulting in an increase in stretch reflexes, and is characterized by excessive tendinous reflexes, significant resistance to passive movement, and hypertonia [8]. Hypertonia is caused by damage to the upper motor neurons resulting from the influence of exaggerated muscle stretching reflexes on tone, causing upper motor neuron syndrome [8,9], and negatively affecting motor performance and quality of life [9]. On the other hand, there is a different phenomenon related to spasticity that results in co-contraction and associated reactions, which do not depend on spinal reflexes, e.g., efferent phenomena and spastic dystonia. Co-contraction is the simultaneous contraction of the agonist and antagonist muscles of a joint. In healthy subjects, an order is issued from the motor cortex that activates the motor neurons to contract the agonist muscles at the same time as a reciprocal inhibition occurs, which consists of the inhibition of the antagonist musculature through the interneurons Ia. When upper motor neuron syndrome occurs, reciprocal inhibition is lost, making it difficult for these individuals to generate strength or movement [10]. The associated reactions correspond to involuntary movements due to the activation of paretic muscles at the time when a voluntary activation of the unaffected muscles occurs, or during the performance of involuntary actions such as yawning, coughing, or sneezing [11]. Spastic dystonia refers to the tonic contraction of one or more resting muscles, producing a relative inability to relax them. This is not caused by muscle stretching, but it is responsive to stretching [12]. This is a relevant characteristic in spastic patients, and is likely related to the prolonged activation of alpha motoneurons in patients with upper motor neuron syndrome [13]. Spasticity has an incidence rate of 4–42.6% in pwS [14]. It has been shown that the suppression of spasticity with botulinum toxin injection results in a decrease in limitations [15]. However, at present, there is no evidence that relieving it improves motor function [16]. Regarding the rehabilitation of these patients, physiotherapists are very important, along with a multidisciplinary team made up of neurologists, nurses, occupational and physical therapists, speech therapists, and social workers, among others, as they use specific techniques to approach treatment from a global perspective [1,17]. These include dry needling [18], long-lasting orthosis [19], kinesiotape [20], transcutaneous electrostimulation [21], and extracorporeal shock waves [22], among others. New technologies have been implemented in recent decades to complement these treatments [23,24].

Stretching (muscle elongation) is currently the most widely used technique in the physical management of spasticity [25]. Its objective is to reduce pain, improve function, maintain or increase the extensibility of soft tissue and joint range of motion (ROM), and normalize muscle tone [25,26]. However, despite its prevalence in rehabilitation, its mechanisms of action remain unclear [27]. Any stretching produces a transient increase in tissue extensibility due to the viscous deformation of the tissue, which dissipates quickly after tissue removal [27,28]. Stretching can be performed manually by therapists, with the help of devices, splints, and molds in series, or through self-stretching [28,29]. The format greatly influences the duration, as manually executed stretching usually lasts a few minutes, whereas stretching with devices can be maintained for days. In the related literature, terms such as static, dynamic, prolonged, and ballistic stretching are often used. In static stretching, there is usually only one repetition, while dynamic stretching involves more than one repetition [25]. An adequate long-term stretching program is needed to reduce spasticity and achieve the proposed objectives. This must take into account the intensity, or amount of constant or varied applied stress, the speed, the amount of repetitions within a session, the duration of stretching in each repetition, the dose, the total time of the final interval, and the frequency or periodicity of the stretching [25]. Some research advises that stretches should be maintained for at least 30 s, with three to four repetitions, five or more times per week [30,31]. It is also essential to know the proper location and position of the structure being stretched and the components that will produce the stretch. For example, stretching the ankle to dorsiflexion with the knee bent applies tension to different structures than when it is performed with the knee stretched [25].

According to recent literature reviews, there are a large number of techniques used to combat spasticity in pwS, such as stretching, dry needling [18], the use of long-term orthoses [19], kinesiotape [20], transcutaneous electrostimulation [21], and extracorporeal shock waves [22], among others. However, there is some uncertainty as to how to apply the different stretching techniques to optimize the results. Some revisions have been published, such as that of Salazar et al. (2019) [32], which deals with the efficacy of positioning in static stretching along with combinations of other techniques and introduces studies with long-lasting orthoses. Another example is that of Sommerfeld et al. (2012) [33], which speaks generally about combined post-stroke spasticity treatments, including stretching. However, none of these tested the effectiveness of different types of stretching alone without them being combined with other techniques. Kerr et al. [19] also deal with this intervention in pwS, but also include the use of long-term orthoses, a factor included in the exclusion criteria of this review. Finally, Bovend’Eerdt et al. [25] reported the effects of stretching in spasticity, but not necessarily post-stroke. However, despite its widespread clinical use, there is no review that specifically describes the relationship between each type of stretching with spasticity. Therefore, this proposed systematic review evaluates the general and specific effects of stretching on spasticity in pwS.

## 2. Materials and Methods

The Preferred Reporting Items for Systematic Review and Meta-Analysis (PRISMA) model [34] was used for this systematic review (List S1). The protocol of this systematic review was registered in the PROSPERO database (CRD42021255768).

### 2.1. Research Strategy

Exhaustive research of studies was conducted up until March 2021 in the following databases: Medline/PubMed, CINAHL, Scopus, Cochrane Library, Web of Science, and Physiotherapy Evidence Database (PEDro).

The research strategy included all available records in any language. The results were filtered to randomized clinical trials (RCT), with no publication deadline. The detailed research strategies in the different databases are shown in Table 1.

### 2.2. Eligibility Criteria

The studies selected for the systematic review were based on the research question following the format of “PICOS” model [35]: Is static/dynamic, passive, or active stretching (I) effective, compared to conventional physiotherapy and/or other intervention, or non-intervention (C) to improve spasticity, ROM, functional evaluation in activities of daily living (ADL), and motor functions, muscular strength and power, gait, risk of fall and pain, and neural and mechanical muscular properties (O) in patients who have suffered a stroke (P)? Study design (S): RCT.

The exclusion criteria were as follows: The use of long-term orthotic devices and the simultaneous combination of stretching with other techniques.

### 2.3. Assessment of the Methodological Quality and Risk of Bias

Regarding the evaluation of methodological quality, in this study, we selected the scale developed by the Physiotherapy Evidence Database (PEDro) [36]. This scale was developed to identify those studies that tend to be valid internally and to redirect clinical decision making. It consists of 11 criteria, granting a point for each criterion met, which are: (1) the selection criteria are described; (2) random assignment; (3) hidden assignment; (4) the groups at the beginning and end were similar; (5) blinded subjects; (6) blinded therapists; (7) blinded evaluators; (8) follow-up of 85% of participants; (9) there are results from both the intervention group and the control even to “intent to treat”; (10) statistical comparisons between groups; (11) point and variability measurements. Criterion (1) influences the external validity of the clinical trial, not the internal validity, so it was not taken into account in the total score. According to the score obtained in this scale, i.e., from 0 to 10, we were able to assess the degree of methodological quality of a certain study. Those with a score of 9–10 points on the PEDro scale were considered to be of excellent methodological quality; those between 6–8 points were considered to be of a good methodological quality; those between 4–5 points were considered to be of a fair methodological quality; and those with less than 4 points were considered to be of a poor methodological quality [36].

The Cochrane collaboration tool [37] was used to analyze the risk of bias in the included studies. It includes six domains: selection bias: random sequence generation and assignment occultation; performance bias: blinding of participants and staff; detection bias: blinding evaluators of the results; wear bias: incomplete result data; reporting bias: selective reporting of results; and other biases. This evaluation comprises three terms: “low risk”, “high risk”, and “unclear risk”, and is presented in a table and a chart.

Two authors (L.G.-C and D.L.-A.) carried out the quality assessment, and an additional reviewer (C.L.-M.) was considered for consensus when needed. The interrater agreement between authors was evaluated with Cohen’s Kappa (K).

### 2.4. Selection Process and Data Extraction

The selection process consists of several stages. Initial research was performed in the main database used, i.e., PubMed, to obtain the most appropriate descriptors. Thereafter, it was implemented in all the databases mentioned, using the strategies shown in Table 1. Once this was carried out, duplicates were removed and the detailed titles and abstracts were read in order to identify studies of great relevance. Finally, we verified those studies that fell within the inclusion criteria that were established for this review. Two authors (L.G.-C and C.L.-M.) carried out the screening process, and an additional reviewer (D.L.-A.) was considered for consensus when needed. The interrater agreement between authors was evaluated with Cohen’s Kappa (K). The data extracted from the selected studies were: Author; year of publication; patient characteristics (total number of participants, number of participants in each group, name of groups to which they belong, age, sex); disease progression time; intervention details, such as type of stretching, sessions/repetitions, follow-up/reviews; variables, including measurement instruments; and results.

### 2.5. Statistical Analysis

A statistical analysis was performed using the Review Manager 5.4 and EPIDAT software version 3.1. This was used to check the homogeneity of the studies and to obtain the results of the meta-analysis. Homogeneity values: I2 > 50%, indicating substantial heterogeneity, wherein randomized effect models were applied, and I2 < 50%, indicating substantial homogeneity, wherein the fixed effect model was applied. The second tool was used to check the risk of publication of the studies selected for meta-analysis. Whenever possible, Begg and Egger values were observed (*p* < 0.05).

In order to provide a complete examination of our primary outcomes—spasticity, measured by the Modified Ashworth Scale (MAS) and Modified Modified Ashworth Scale (MMAS), and ROM—two different analyses were performed, with a 95% confidence interval. *p* values ≤ 0.05 were considered statistically significant.

## 3. Results

**Figure 1:**
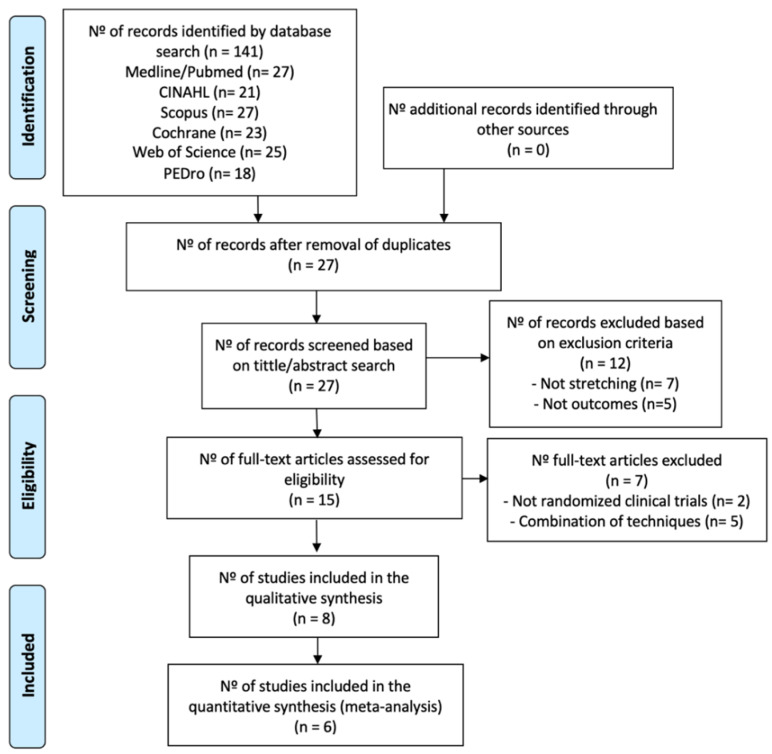
Information flowchart of the different phases of the systematic review.

Of a total of 141 studies identified, only eight studies of great relevance were selected for this systematic review (Figure 1). A list was made to show the items that were excluded and the reasons for the exclusion, which can be found in the Supplementary Materials (List S2).

### 3.1. Methodological Quality and Risk of Bias

The PEDro scores achieved by each of the studies selected for this review are shown in Table 2. For each of them, the score shown in the PEDro database was collected, excepting two studies [38,39] in which the evaluation was performed manually. The overall score was 5.5 on average, denoting a moderate/good methodological quality of the selected studies, with scores ranging from 4 [40,41] to 8 [39].

Regarding the risk of bias, the authors Pradhan and Bansal [40], Ghasemi et al. [38] and Carda et al. [42] presented the lowest risk of bias. Kim et al. [43] presented the highest risk of bias.

**Table 2:** Score obtained on the PEDro scale for each of the selected studies.

Study	Total	1	2	3	4	5	6	7	8	9	10	11
**Pradhan and Bansal 2018** [40]	4/10	-	0	0	1	0	0	1	0	0	1	1
**Ghasemi et al. 2018** [44]	5/10	-	1	0	1	0	0	1	0	0	1	1
**Ghasemi et al. 2018** [38]	6/10	-	1	0	1	0	0	1	1	0	1	1
**Jang et al. 2016** [45]	5/10	-	1	0	1	0	0	1	0	0	1	1
**Santamato et al. 2015** [39]	8/10	-	1	1	1	0	0	1	1	1	1	1
**Kim et al. 2013** [43]	5/10	-	1	0	1	0	0	0	1	0	1	1
**Jung et al. 2011** [41]	4/10	-	1	0	1	0	0	0	0	0	1	1
**Carda et al. 2011** [42]	7/10	-	1	1	1	0	0	1	1	0	1	1

**Figure 2:**
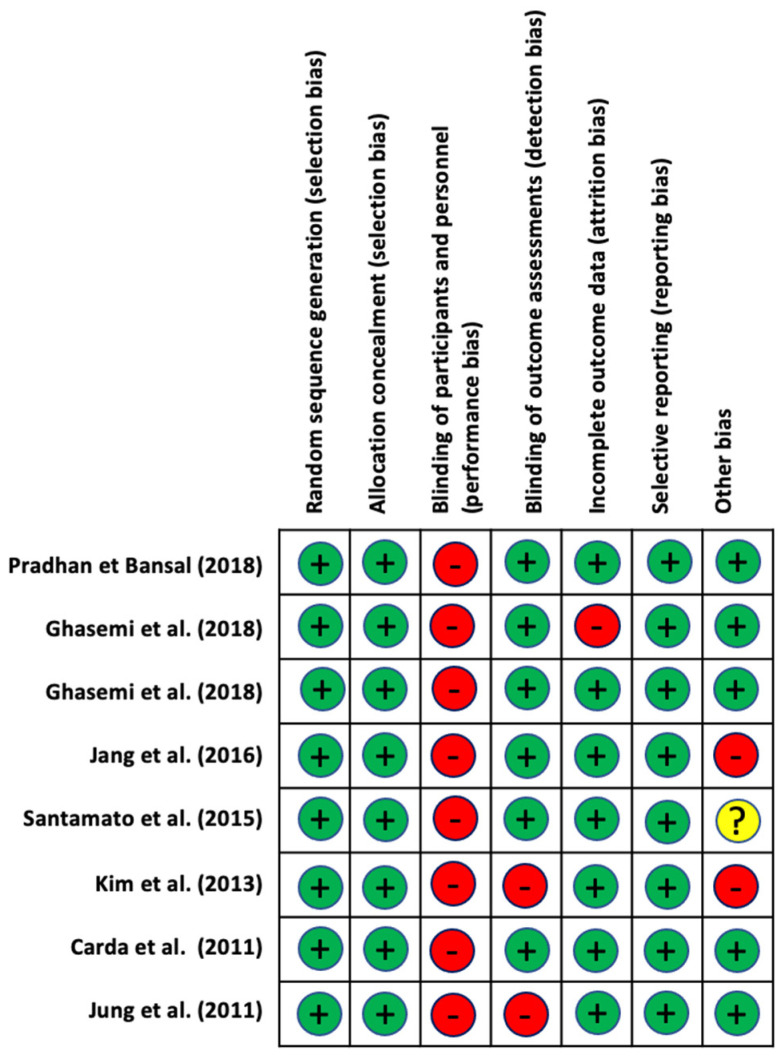
Risk of bias of the studies included in the systematic review.

**Figure 3:**
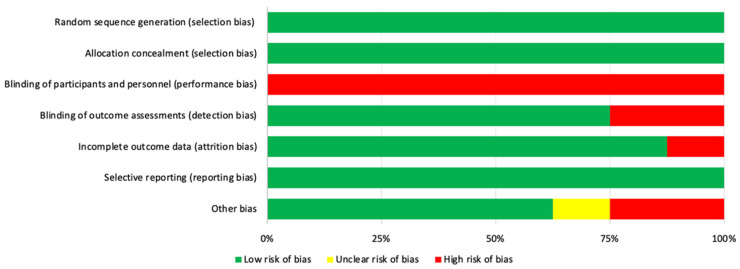
Overall risk of bias. The results are presented by percentages.

With respect to the evaluation of the different types of biases according to the selected studies, we can see that the items that obtained lower biases were the generation of the random sequence (selection bias), the occultation of the assignment (selection bias), and selective reporting of results (reporting bias). On the other hand, the highest value was given for the blinding of participants and staff (performance bias). The results are shown in Figure 2 and Figure 3.

### 3.2. Synthesis of Results

In Table 3, we can see the most relevant characteristics of the included studies.

### 3.3. Participant Characteristics

A total of 332 pwS with spasticity were evaluated from the eight studies selected for this review. The average age of individuals was 53 years. At the time of intervention, the patients had a chronic stroke with spasticity 3–6 months post-stroke, except for the patients in the Pradhan and Bansal study [40], who were at least 6 weeks post-stroke. Moreover, Ghasemi et al. [38] indicated that the patients suffered a chronic stroke, but did not specify the exact time.

### 3.4. Intervention Characteristics

The stretches used varied depending on whether they were performed manually by the physiotherapist [40,42], involved self-stretching [38,44], or utilized external help, such as devices [39,41,43,45]. In the case of Santamato et al. [39], they only used an external aid when providing static passive stretching, as they combined two types of stretching. In the studies in which stretching was performed manually, it was carried out by the physiotherapist [40,42] or the patient themselves, with a wedge or inclined plane as an aid [38,44].

Moreover, we were able to classify the studies depending on whether passive static stretching [38,43,44,45] or passive dynamic stretching [40,41,42] was performed as an intervention. Finally, Santamato et al. [39] combined prolonged static (splint) and dynamic static passive stretching in their intervention.

The duration of the intervention varied greatly between studies, but none of them exceeded 60 min/session. In terms of follow-up studies, three evaluations were generally performed: At the beginning, after treatment, and a long-term follow-up.

### 3.5. Study Groups Included in the Statistical Analysis

**Figure 4:**
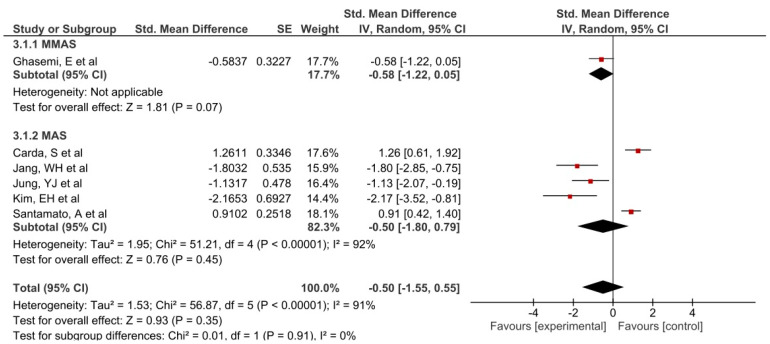
Forest plot of the comparison of the difference between the subgroups in MAS/MMAS.

**Figure 5:**
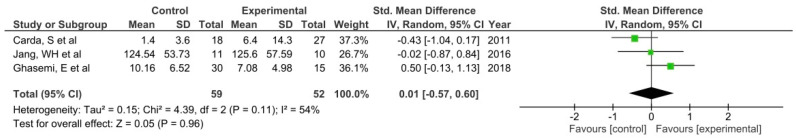
Forest plot of the comparison of the difference between the groups in ROM.

Of all the outcomes extracted from the studies included in this research, only two were included in the meta-analysis (Figure 4 and Figure 5). The results of this meta-analysis indicate that there was heterogeneity in muscle tone for passive movement, measured by the MAS and MMAS, and ROM (I^2^ > 50%).

**Figure 6:**
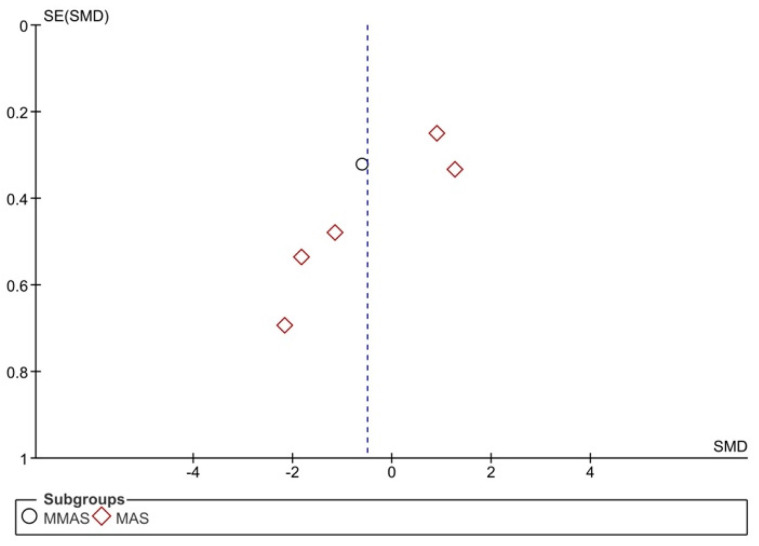
Funnel plot panel of the comparison of the difference between the subgroups in MAS/MMAS.

**Figure 7:**
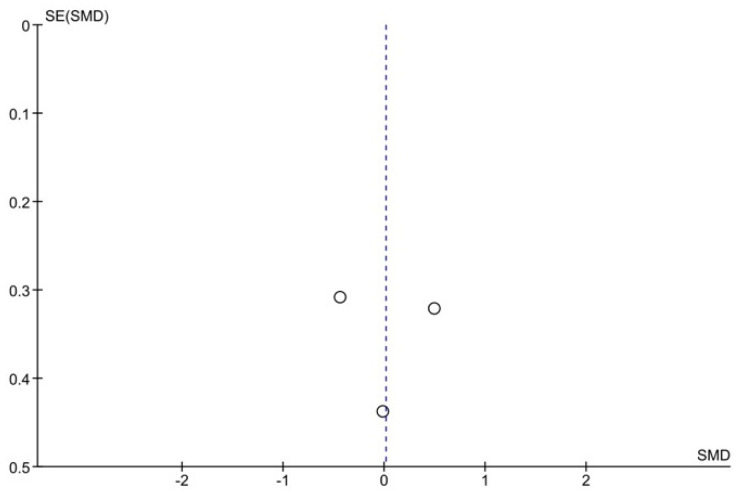
Funnel plot panel of the comparison of the difference between the groups in ROM.

There was no publication bias for MAS/MMAS (Begg’s test: *p* = 1.00; Egger’s test: *p* = 0.65) and ROM (Begg’s test: *p* = 0.29; Egger’s test: *p* = 0.37) outcomes, but these data should be treated with caution because the number of studies included in the analysis is low (Figure 6 and Figure 7).

#### Spasticity

This variable is the only measurement that exists in all of the studies. The standard scale used was the MAS [46], with scores in the range of 0–4: (0) no increase in muscle tone; (1) a slight increase in muscle tone, manifested by a block and release or by a minimum resistance at the end of the ROM when the affected segment moves in flexion or extension; (2) increased muscle tone, demonstrated by a block in the middle of the ROM with effortless movement of the affected party; (3) considerable increase in muscle tone, difficult passive movement; (4) rigid parts affected in flexion or extension. This is the most widely used scale, although there is no guide to standardize its use and it does not relate posture to tone [46].

Non-statistically significant results in favor of the intervention group were observed for MAS (Std.MD subtotal = −0.50 (−1.55, 0.55)). The best results were obtained by Jang et al., Jung et al. [41], and Kim et al. [43] for the stretching group, and Carda et al. [42] and Santamato et al. [39] for the control group.

### 3.6. Range of Motion

Three studies [42,44,45] were included in this analysis group, with controversial results. Results were not conclusive for the ROM outcome (Std. MD = 0.01 [−0.57, 0.60]).

### 3.7. Other Outcomes

#### 3.7.1. Activities of Daily Living and Motor Functions

For this variable, which was evaluated by three authors [39,40,45], Pradhan and Bansal [40] used the Modified Rankin Scale (mRS) and the Barthel index and obtained a significantly greater improvement especially at 6 months. Jang et al. [45] used the Fugl-Meyer Assessment (FMA) to evaluate motor functions, which led to significant results. Finally, Santamato et al. [39] used the Disability Assessment Scale (DAS), maintaining pain relief as a primary objective to assess disability in the upper limb of its participants, but they did not obtain significant results.

#### 3.7.2. Muscle Strength

These are presented in two studies [40,42]. Pradhan and Bansal [40] used the Modified Medical Research Council scale (mMRC), significantly improving the results obtained for the upper and lower limb from the beginning of the procedure. However, for the strength evaluation, Carda et al. [42] did not obtain significant improvements in the ankle and foot muscles using the Medical Research Council scale (MRC).

#### 3.7.3. Gait

This variable was only evaluated in a studio [42]. Carda et al. [42] assessed the gait with three measuring instruments: 6-Minute Walking Test (6MWT), 10-Meter Walking Test (10MWT), and Functional Ambulation Category (FCA). They measured gait speed and endurance, gait speed, and wandering ability, respectively. With 6MWT and FCA, no improvements were obtained. However, with 10MWT, nonsignificant improvements were reported.

#### 3.7.4. Risk of Fall and Pain

These variables were evaluated by Ghasemi et al. [38] with Timed Up and Go (TUG) for fall risk, and the Visual Analogue Scale (VAS) for pain. Statistically significant improvements were reported.

#### 3.7.5. Neural and Mechanical Properties

This variable was specifically studied by Ghasemi et al. [44] and was measured through electromyography and an ultrasound. They observed significant improvements with respect to the comparative group in the penance angle and muscle thickness. Furthermore, significant differences were reported for H-reflex latency after a follow-up of the experimental group.

## 4. Discussion

The results of this meta-analysis showed heterogeneity in increase in muscle tone for passive movement (MAS/MMAS) and ROM (I^2^ > 50%).

The passive stretches analyzed in the selected articles (static and dynamic) produced an improvement in at least some of the variables assessed. However, not all of them returned significant results demonstrating their effectiveness. The efficiency achieved with passive static stretching may result from the few limits initially presented, or its efficiency with respect to others. However, studies involving passive static stretching used a protocol in which stretching is kept relatively short. One factor that can influence the results is the mode of application of stretching. The interventions that required a physical therapist for stretching took less time than the interventions involving self-stretching [38,44] or external aid [39,41,43,45]. It is possible that interventions involving means such as long-lasting orthoses or splints to prolong the effects of stretching over time were even better than a short-lived static passive or dynamic stretching.

In the meta-analysis of Salazar et al. [32] the results were positive for passive static stretching, including long-term orthoses, as an isolated intervention compared to nonintervention. Another related review is that of Bovend’Eerdt et al. [25], in which no clear conclusions on stretching were drawn because of the study’s limitations. However, it evaluates the effects of stretching on spasticity, but includes conditions such as multiple sclerosis and brain damage.

Passive stretching presents potential benefits in pwS with spasticity, although the intervention should be individualized and adapted to each patient and situation by the therapist, measuring both the duration of the stretching and the repetitions and sessions. A heterogeneity was identified in the outcome, intervention, and methodology measures of the selected studies that meet the inclusion criteria, so the results could be evaluated based on the following variables.

### 4.1. Spasticity

Spasticity, the most common complication associated with strokes, is the main variable revealed in this review. All of the studies evaluated this outcome. Carda et al. [42] and Jang et al. [45] did not achieve significant results in this regard, possibly due to its short implementation time compared to the other groups’ interventions. Santamato et al. [39] found significant differences in favor of the control group (taping), in line with what was reported in the literature [20,47] (the combination with botulinum toxin in both groups could have affected the results). It would be interesting to assess the isolated effects of stretching and its combination with taping for improving post-stroke spasticity.

Performing passive stretching periodically is extremely important, as stretching is known to reduce the spasticity of the affected joints; however, it returns after some time [40]. Pradhan and Bansal [40] stated that the intervals between repetitions should be 2.5–3 h so that pain does not occur, so the need to perform multiple repetitions at frequent intervals is emphasized. In turn, it would be necessary to carry out a program that is sufficiently long, with respective evaluations before, during, after, and in a post-intervention period, to evaluate effectiveness in both the short and the long term. The previous authors also stated that stretching involved certain important factors such as speed, pain, and the position of the affected segment, which may influence the management of spasticity. In this sense, choosing the appropriate type of stretching is essential; thus, individualizing the intended physiological effects depending on the personalized alterations in each type of patient (e.g., myotatic reflex, inverse myotatic reflex).

### 4.2. Range of Motion

This outcome, measured in four studies [38,42,44,45], has two subtypes: Active and passive ROM, which yielded inconclusive results.

Carda et al. [42] measured the passive ROM, comparing the efficacy of three interventions: splints, adhesive tape, and passive stretching, after administering a dose of BTX-A in the three groups. The administration of this toxin was not involved in the comparison between groups because the same dose was given to the three groups at the beginning of the intervention. The results demonstrate that the splint-using group was the only group that obtained significant improvements until the follow-up, which was not the case for the group that performed dynamic passive stretching. This poor result is especially striking because this is a commonly prescribed treatment after botulinum toxin type A in patients with spasticity. One possible explanation is that the stretching was applied for 1 h a day, whereas the other two techniques were maintained for 24 h a day. In addition, the authors suggested that prolonged stretching can improve the internalization of botulinum toxin type A, producing muscle activation with elicitation of tonic stretching reflex and generating a positive effect on the viscoelastic properties of spastic muscles. On the other hand, the studies by Ghasemi et al. [38,44] achieved potential improvements, eventually leading to an improved gait.

In the case of active ROM, Jang et al. [45] did not achieve significant motor recovery, although the measured values increased over time in the intervention group, without a force evaluation.

### 4.3. Other Outcomes

#### 4.3.1. Daily Life Activities and Motor Functions

These outcomes were evaluated using different measurement instruments in each of the studies [39,40,45].

Jang et al. [45] achieved a relief from spasticity along with a functional recovery of the hand and wrist using the passive muscle stretching device for 4 weeks. There was a wide variety of studies investigating the use of passive stretching devices for lower limb spasticity [48,49,50], but it was more difficult to find those dealing with wrist and hand devices. In 2011, Brokaw et al. [51] developed a stretching device for extending the wrist by means of elastic cords in pwS; great benefits were reported in the functionality of the hand and the ROM. Pradhan and Bansal [40] did not achieve significant improvements in the Barthel index at 3 months. However, in the evaluation carried out at 6 months, they achieved significant results, reporting physical improvements. Santamato et al. [39] compared two intervention groups: using kinesiotape and performing manual passive stretching, which consisted of static passive stretching with a splint for 30 min after 30 min of manual dynamic passive stretching. In addition to stretching, a dose of BTX-A was also injected into subjects in both groups. The administration of this toxin was not involved in the comparison between groups because the same dose was given to the two groups at the beginning of the intervention. The group using the kinesiotape obtained significant improvements in DAS with respect to the group performing manual stretching. Similarly, these results may result from the time of application of the intervention, as the kinesiotape was used for 10 consecutive days, and the performance of the stretch was maintained for only 60 min per day.

#### 4.3.2. Muscle Strength

Strength was measured in two studies [40,42] by the MRC and the mMRC, respectively. Pradhan and Bansal7 [40] analyzed two stretching techniques, but one of them, specifically the Corrected Assisted Synchronized Periodic (CASP) technique, included active synchronized patient exertion over the full range. The results of this study demonstrate that CASP therapy produced a significant improvement as compared to conventional therapy. From these results, we deduced that active exertion on the full ROM after stretching, which is the main way in which the two techniques differ, could be favorable in terms of improving muscle power, spasticity, and the functional evaluation of ADL in these types of patients. It should be emphasized that the correction of the position of the affected element is an aspect that CASP therapy highly values. Furthermore, it places emphasis on the role of assistance and synchronization, as it ensures that the full ROM is completed with the patient’s mind working both at the cognitive and psychological level. This full ROM ensures that contractures do not develop, and that the disability related to contracture is minimized [40]. Finally, in the study by Carda et al. [42], muscle strength did not yield favorable results, unlike the other variables under study.

#### 4.3.3. Gait

Carda et al. [42] did not achieve favorable results on the road test despite the variety of measuring instruments used. In this case, only 60-minute sessions were conducted over a week, with the treatment time being a key factor for obtaining good results. These observations are in line with those of Bressel and McNair [52], who reported that a 30-minute stretch of the ankle plantar flexors reduced stiffness in the ankle joint, but had no effect on the gait. Plastic changes in the central nervous system develop after repeated training, which are necessary in order to enable adaptation to the demands of any motor task [53]. It is, therefore, understandable that few muscle stretching sessions have little or no effect on the functional plasticity of the neural circuits of the spinal cord [54]. Therefore, to improve the spastic gait in pwS, it is necessary to establish a muscle stretching program that has repeated treatment sessions over several days [42].

#### 4.3.4. Risk of Fall and Pain

Ghasemi et al. [38] placed great importance on these variables. According to Wu et al. [55], obtaining significant results in both may be largely due to the reduction in spasticity that comes with the technique, which leads to greater performance in running and performing the activities of daily life.

According to Ng and Hui-Chan [56], increased TUG values are dependent on the symptoms of stroke, including muscle weakness and spasticity. Moreover, muscle limitation and spasticity are due to the individual’s inability to perform a functional task with adequate muscle strength. This may, therefore, explain the long time that it took to perform the TUG test.

#### 4.3.5. Neural and Mechanical Properties

In the study by Ghasemi et al. [44], in addition to measuring standard variables, they evaluated neural and mechanical properties, obtaining significant data. The results of this study show that performing a stretching program with functional exercises increases the Hmax/Mmax ratio and reduces the H-reflex latency, and these changes are maintained for at least 2 months. There are different studies that contradict these results, such as those of Guissard and Duchateau [57], which show that performing a passive static stretching program decreased the Hmax/Mmax ratio in healthy subjects. Moreover, Tsai et al. [58] revealed that a single session of prolonged muscle stretching can decrease the Hmax/Mmax ratio in pwS with spasticity. Finally, in another study [59], there were no significant changes in the amplitude and H-reflex latency. Ghasemi et al. [44] reported positive results after a functional intervention, but not dynamic passive stretching, was performed—a factor influencing voluntary motor activities.

### 4.4. Study Limitations and Future Research Lines

This systematic review and meta-analysis have some limitations. The way in which results were reported by the studies made it impossible to include more clinical trials in the meta-analysis. It would be necessary for the RCTs to provide results with standard deviations. Another limitation is related to the high heterogeneity found in the meta-analysis, so the results should be treated with caution.

The choice of studies in which the intervention was combined with BTX-A represents another limitation [39,42]. This factor did not influence the results as the treatment was applied in all groups at the beginning of the intervention, but it did have an impact on the comparative analysis of the results of the different studies selected. Another limitation is the inclusion of studies in which the intervention was very short in duration [42], and those that combined two types of stretching in the same experimental group [39]. There are differences in the minimum duration of the disease; there is even a study [38] in which these data do not directly appear. Long-term evaluations are important, which were lacking in some of the selected studies [41,43].

It is advisable to develop RCTs with a low risk of bias that do not present combinations of techniques, and that include long-term interventions and an adequate sample size, analyzing by means of stratified studies the effects obtained in the different stroke phases. The additional effect of stretching is interesting, particularly in terms of optimizing the other techniques. Currently, there is research being conducted on the nonpharmacological treatment of spasticity in pwS, which focuses on combinations of techniques and global techniques that achieve truly positive results [60,61]. However, it is also important to assess how each of the techniques perform individually, thus allowing for more personalized interventions. Future studies should focus on analyzing the impact of the active components of spasticity during movements, and the influence of active stretching on spasticity reduction. Future research should continue evaluating the neural and mechanical properties of muscles, and the implementation of electrophysiological evaluations that assess the real impact of these techniques.

## 5. Conclusions

There is no conclusive evidence on the effectiveness of stretching interventions for improving spasticity and ROM in pwS. However, for the improvement spasticity, it is advisable to perform passive stretching for a prolonged period. This technique produced better results than techniques involving few repetitions for a short period of time. Similarly, it would be useful to analyze the active components of spasticity, i.e., those that are not evaluated by the MAS scale. Despite limited evidence, passive static stretching is a widely used technique in reducing post-stroke spasticity. It demonstrated potential benefits not only in terms of spasticity, but in other variables conditioned by it. Regarding functionality in the activities of daily life, sufficiently positive results were obtained to warrant further investigations into its parameters. Positive results were also reported in other outcomes evaluated in single studies, e.g., associated with gait, the risk of fall and pain, and mechanical and neural properties. Future studies should focus on these. In terms of ROM, the results were heterogeneous; therefore, more research is needed to draw reliable conclusions. Passive stretching, along with active synchronized exertion, appears to offer great benefits in terms of strength. However, it is not effective for these variables if a program that includes only stretching is followed.

Further research is necessary to determine the effectiveness of stretching in this population. It should consider the different types of stretching (static and dynamic), the time of application, the measurement of the different components of spasticity, and the extrapolation of functional results.

## Figures and Tables

**Table 1:** Research strategy for the different databases.

Databases	Search
Medline/PubMed, CINAHL, Scopus, Cochrane, Web of Science	(“stretch” OR “stretching” OR “stretching exercises” OR “stretching flexibility” OR “mobility” OR “flexibility” OR “range of motion” OR “muscle stretching exercises” OR “passive motion” OR “assisted movement” OR “constraint-induced movement” OR “tissue expansion” OR “tissue expansion devices”) AND (“spasticity” OR “muscle spasticity” OR “pyramidal hypertonia” OR “muscle rigidity” OR “muscle hypertonia”) AND [“stroke”]
PEDro	Stretch * spasticity * stroke *

*, The asterisk character performed a multiple character search. For example, the search term Stretch* retrieved results such as stretching, stretched, and so on.

**Table 3:** Synthesis of Results.

Authors (Year)	Sample	Type of Stretching	Age (Average)	Stadium	N Sessions Temporality	Performance of Measurement	Results
**Pradhan et Bansal** (2018) [40]	N = 61 CASP = 31 CT = 30	CASP = Passive dynamic stretching + active effort TC = Passive dynamic repetitive stretching	51.15	Chronicity +6 months	30 min/session. 6 months. CASP = 6 sessions/day. TC = 2–3 sessions/day.	MAS (0–4), mMRC (0–10), mRS (0–6), Barthel index (0–100)	A significantly greater improvement was obtained in CASP therapy (*p* < 0.001). However, both improved in all the variables studied.
**Ghasemi et al.** (2018) [44]	N = 45 EG = 30 CT = 15	Passive static stretching + functional stretching exercises	56.25	Chronicity +6 weeks	30 s/stretch. 3 sessions/week. 4 weeks.	EMG, ultrasound machine. MMAS (0–4), reflex hammer (0–4), goniometer.	Significant improvements in EG tracking were in the angle of penetration (*p* = 0.006) and muscle thickness (*p* = 0.030).
**Ghasemi et al.** (2018) [38]	N = 30 EG = 15 CT = 15	Passive static stretching + functional stretching exercises	52.27	Chronicity +3 months	30 s/stretch. 3 sessions/week. 4 weeks.	MMAS (0–4), reflex hammer (0–4), goniometer. 10 m WTT, TUG, VAS (0–10)	The comparison between the two groups showed significant differences only in spasticity (MMAS *p* = 0.048) and pain (VAS *p* = 0.001).
**Jang et al.** (2016) [45]	N = 21 IG = 11 CG = 10	Passive static stretching with dispositive	49.1 ± 13.5	Chronic	4 min/stretch. 3 stretch/session. 3 sessions/day. 6 days/week. 4 weeks.	MAS, FMA, goniometer	Significant improvements were obtained in the IG in spasticity (MAS) and motor functions (FMA), both observing its follow-up and comparing with the CG (*p* < 0.05).
**Santamato et al.** (2015) [39]	N = 70 Taping + BTX-A = 35 Stretching + BTX-A = 35	Passive dynamic stretching + static stretching with splint	56.9	Chronicity +6 months	30 min/session passive dinamic. 30 min/session passive static. 10 days.	MAS (0–4), DAS (0–3)	After two weeks, “taping” obtained a significant reduction in spasticity (MAS *p* < 0.01), and after one month, it was added the decrease in disability in the upper limb (*p* < 0.01) compared to “stretching”.
**Kim et al.** (2013) [43]	N = 15 IG = 8 CG = 7	Passive static stretching with dispositive	51.2 ± 11.4	Chronicity +6 months	10 min/session. 2 sessions/day. 4 weeks.	MAS (0–4)	Comparing both groups, the IG showed a significant decrease in spasticity after treatment (*p* < 0.001).
**Jung et al.** (2011) [41]	N = 21 IG = 10 CG = 11	Passive dynamic stretching with dispositive	46.6 ± 10.9	Chronicity +6 months	30 s/stretch. 20 min/session. 2 sessions/day. 6 days/week. 3 weeks.	MAS (0–4)	The IG showed significant improvement during treatment with respect to CG (*p* < 0.001), but after a week after treatment, spasticity increased again.
**Carda et al.** (2011) [42]	N = 69 Taping + BTX-A = 24 Casting + BTX-A = 27 Stretching + BTX-A = 18	Passive dynamic stretching	62.1	Chronicity +6 months	30 min/session. 2 sessions/day. 1 week.	MAS, goniometer, 6MWT, 10MWT, FCA, MRC	The comparison between the three groups showed no significant differences (*p* > 0.05). However, the follow-up of the Stretching group showed improvements in spasticity, PROM, speed, and endurance in the walk.

BTX-A, Botulinum Toxin type A; CASP, Corrected-Assisted-Synchronized-Periodic; CG, Control Group; CT, Conventional Therapy; DAS, Disability Assessment Scale; EG, Experimental Group; EMG, Electromiography; FCA, Functional Ambulation Category; FMA, Fugl-Meyer Assessment; IG, Intervention Group; MAS, Modified Ashworth Scale; MMAS, Modified Modified Ashworth Scale; mMRC, Modified Medical Research Council scale; mRS, Modified Rankin Scale; MRC, Medical Research Council scale; TUG, Timed Up and Go; VAS, Visual Analogue Scale; 6MWT, 6-Minute Walking Test; 10 m WTT, 10 m Walking Timed Test; 10MWT, 10-Meter Walking Test.

## Supplementary Materials

The following are available online at https://www.mdpi.com/article/10.3390/jpm11111074/s1, List S1: PRISMA checklist; List S2: Excluded articles and reasons.
