# Supplementary material for: Effectiveness of Stretching in Post-Stroke Spasticity and Range of Motion: Systematic Review and Meta-Analysis
Source: J Pers Med. 2021 Oct 24;11(11):1074. doi: 10.3390/jpm11111074 (PMC8619362; doi:10.3390/jpm11111074)
Supplement: Supplementary file 1 [file jpm-11-01074-s001.zip › List S2.pdf]

List S2: Excluded articles and reasons.

EXCLUDED ARTICLES BASED ON EXCLUSION CRITERIA (N=12)

Not stretching (n=7)

1. Hu, X.L.; Tong, K.Y.; Song, R.; Zheng, X.J.; Leung, W.W. A Comparison Between Electromyography-Driven Robot and Passive Motion Device on Wrist Rehabilitation for Chronic Stroke. *Neurorehabil. Neural Repair* **2009**, *23*, 837–846, doi:10.1177/1545968309338191.
2. Nasb, M.; Li, Z.; S.A. Youssef, A.; Dayoub, L.; Chen, H. Comparison of the effects of modified constraint-induced movement therapy and intensive conventional therapy with a botulinum-a toxin injection on upper limb motor function recovery in patients with stroke. *Libyan J. Med.* **2019**, *14*, 1609304, doi:10.1080/19932820.2019.1609304.
3. Chen, S.; Lv, C.; Wu, J.; Zhou, C.; Shui, X.; Wang, Y. Effectiveness of a home-based exercise program among patients with lower limb spasticity post-stroke: A randomized controlled trial. *Asian Nurs. Res. (Korean. Soc. Nurs. Sci).* **2020**, *15*, 1–7, doi:10.1016/j.anr.2020.08.007.
4. Fleuren, J.F.; Nederhand, M.J.; Hermens, H.J. Influence of Posture and Muscle Length on Stretch Reflex Activity in Poststroke Patients With Spasticity. *Arch. Phys. Med. Rehabil.* **2006**, *87*, 981–988, doi:10.1016/j.apmr.2006.03.018.
5. DeMeyer, L.; Brown, M.; Adams, A. Effectiveness of a night positioning programme on ankle range of motion in patients after hemiparesis: a prospective randomized controlled pilot study. *J Rehabil Med* **2015**, *47*, 873–877, doi:10.2340/16501977-2007.
6. Nelson, D.L.; Konosky, K.; Fleharty, K.; Webb, R.; Newer, K.; Hazboun, V.P.; Fontane, C.; Licht, B.C. The effects of an occupationally embedded exercise on bilaterally assisted supination in persons with hemiplegia. *Am. J. Occup. Ther.* **1996**, *50*, 639–646, doi:10.5014/ajot.50.8.639.
7. Carey, J.R. Manual stretch: Effect on finger movement control and force control in stroke subjects with spastic extrinsic finger flexor muscles. *Arch. Phys. Med. Rehabil.* **1990**, *71*, 888–894.

Not outcomes (study of excluded variables) (n=5)

8. Fischer, H.C.; Stubblefield, K.; Kline, T.; Luo, X.; Kenyon, R. V.; Kamper, D.G. Hand rehabilitation following stroke: A pilot study of assisted finger extension training in a virtual environment. *Top. Stroke Rehabil.* **2007**, *14*, 1–12, doi:10.1310/tsr1401-1.
9. Bakheit, A.M.; Maynard, V.; Shaw, S. The effects of isotonic and isokinetic muscle stretch

on the excitability of the spinal alpha motor neurones in patients with muscle spasticity. *Eur. J. Neurol.* **2005**, *12*, 719–724, doi:10.1111/j.1468-1331.2005.01068.x.

10. Maynard, V.; Bakheit, A.M.; Shaw, S. Comparison of the impact of a single session of isokinetic or isotonic muscle stretch on gait in patients with spastic hemiparesis. *Clin. Rehabil.* **2005**, *19*, 146–154, doi:10.1191/0269215505cr853oa.
11. Rattes, C.; Campos, S.L.; Morais, C.; Gonçalves, T.; Sayão, L.B.; Galindo-Filho, V.C.; Parreira, V.; Aliverti, A.; Dornelas de Andrade, A. Respiratory muscles stretching acutely increases expansion in hemiparetic chest wall. *Respir. Physiol. Neurobiol.* **2018**, *254*, 16–22, doi:10.1016/j.resp.2018.03.015.
12. Wolf, S.L.; Segal, R.L.; Catlin, P.A.; Tschorn, J.; Raleigh, T.; Kontos, H.; Pate, P. Determining consistency of elbow joint threshold angle in elbow flexor muscles with spastic hypertonia. *Phys. Ther.* **1996**, *76*, 586–600, doi:10.1093/ptj/76.6.586.

FULL-TEXT ARTICLES EXCLUDED (N=7)

Not randomized clinical trials (n=2)

13. Salazar, A.P.; Pinto, C.; Ruschel Mossi, J.V.; Figueiro, B.; Lukrafka, J.L.; Pagnussat, A.S. Effectiveness of static stretching positioning on post-stroke upper-limb spasticity and mobility: Systematic review with meta-analysis. *Ann. Phys. Rehabil. Med.* **2019**, *62*, 274–282, doi:10.1016/j.rehab.2018.11.004.
14. Jo, H.M.; Song, J.C.; Jang, S.H. Improvements in spasticity and motor function using a static stretching device for people with chronic hemiparesis following stroke. *NeuroRehabilitation* **2013**, *32*, 369–375, doi:10.3233/NRE-130857.

Combination of techniques (n=5)

15. Yamaguchi, T.; Tanabe, S.; Muraoka, Y.; Masakado, Y.; Kimura, A.; Tsuji, T.; Liu, M. Immediate effects of electrical stimulation combined with passive locomotion-like movement on gait velocity and spasticity in persons with hemiparetic stroke: A randomized controlled study. *Clin. Rehabil.* **2012**, *26*, 619–628, doi:10.1177/0269215511426803.
16. Waldman, G.; Yang, C.Y.; Ren, Y.; Liu, L.; Guo, X.; Harvey, R.L.; Roth, E.J.; Zhang, L.Q. Effects of robot-guided passive stretching and active movement training of ankle and mobility impairments in stroke. *NeuroRehabilitation* **2013**, *32*, 625–634, doi:10.3233/NRE-130885.
17. Ribeiro, T.; Britto, H.; Oliveira, D.; Silva, E.; Galvão, E.; Lindquist, A. Effects of treadmill

training with partial body weight support and the proprioceptive neuromuscular facilitation method on hemiparetic gait: A randomized controlled study. *Eur. J. Phys. Rehabil. Med.* **2013**, *49*, 451–61.

18. De Jong, L.D.; Dijkstra, P.U.; Gerritsen, J.; Geurts, A.C.; Postema, K. Combined arm stretch positioning and neuromuscular electrical stimulation during rehabilitation does not improve range of motion, shoulder pain or function in patients after stroke: A randomised trial. *J. Physiother.* **2013**, *59*, 245–254, doi:10.1016/S1836-9553(13)70201-7.
19. Etoh, S.; Noma, T.; Miyata, R.; Shimodozono, M. Effects of Repetitive Facilitative Exercise on Spasticity in the Upper Paretic Limb After Subacute Stroke. *J. Stroke Cerebrovasc. Dis.* **2018**, *27*, 2863–2868, doi:10.1016/j.jstrokecerebrovasdis.2018.06.013.
